# Are Štampar’s principles valid in the light of the Sustainable Development Goals?

**DOI:** 10.3325/cmj.2020.61.173

**Published:** 2020-04

**Authors:** Ana Borovečki, Selma Šogorić, Mirjana Kujundžić Tiljak

**Affiliations:** 1 Department of Social Medicine and Organization of Health Care, Andrija Štampar School of Public Health, University of Zagreb School of Medicine, Zagreb, Croatia *ana.borovecki@mef.hr*; 2 Department of Medical Statistics, Epidemiology and Medical Informatics, Andrija Štampar School of Public Health, University of Zagreb School of Medicine, Zagreb, Croatia

The 2030 Agenda for Sustainable Development, adopted by all United Nations Member States in 2015, defines 17 Sustainable Development Goals (SDGs) (1). There is a close link between SDGs and another concept championed by the United Nations: WHO – Social Determinants of Health (SDH) (2), incorporated into the WHO Health 2020 policy framework (3) (Table 1). The synergy and obvious similarity between the aims stated in both documents guided the WHO decision to introduce the SDGs into their future strategies. The European Commission created the *Proposal for a New European Consensus on Development – Our World, our Dignity, our Futur*e (4,5), setting new agenda in the light of global challenges and the AGENDA 2030 by the United Nations. The document clearly recognizes the SDG:

**Table 1:** The similarities between Sustainable Developmental Goals and Social Determinants of Health

Sustainable Development Goals	Social Determinants of Health
End poverty Reduce inequality within and among countries	The social gradient (income and income distribution)
Ensure healthy lives and promote well-being for all at all ages	Early childhood development stress addiction
Ensure inclusive and equitable quality education and promote lifelong learning opportunities for all	Education
Enforce gender equality	Social exclusion (gender, race, disability)
Create decent work and sustainable economic growth	Work (unemployment and job security employment and working conditions)
End hunger Ensure sustainable consumption and production patterns Ensure availability and sustainable management of water and sanitation for all Ensure access to affordable and clean energy Conserve and sustainably use the oceans, seas and marine resources for sustainable development Protect, restore and promote sustainable use of terrestrial ecosystems Sustainably manage forests Combat desertification Halt and reverse land degradation Halt biodiversity loss Make cities and human settlements inclusive, safe, resilient and sustainable Build resilient infrastructure Promote inclusive and sustainable industrialization Foster innovation Take urgent actions to combat climate change	Food (insecurity) Physical environment Built (urban) environment Housing Transportation
Promote peaceful and inclusive societies for sustainable development Provide access to justice for all Build effective, accountable and inclusive institutions at all levels Build partnerships for the goals	Social environment (social support, social safety network, health services)

“*1. The world has changed considerably since the last European Consensus on Development in 2005, in terms of both opportunities and risks.*

2. The levels and geography of poverty and inequality have shifted and developing countries have become increasingly diversified.

3. Building resilience and sustainability is indispensable for lasting solutions to complex global challenges.

4. Global public goods are under stress because of environmental problems, in particular the challenge of climate change, which threatens development gains and disproportionately affect the poor as well as access to sustainable and affordable energy services.

*5. The development landscape is expanding, encompassing more and new actors and innovative solutions*.”

However, long before the SDGs and SDH there were 10 principles of Andrija Štampar (1888-1958), which were presented publicly in 1926 (Box 1). Štampar was a visionary who would later become one of the founders of the WHO (6,7). The principles were written at the time when health literacy was low, the majority of the population lived in rural areas, and infectious disease and socio-medical problems were the main focus of public health agenda, but even today they show similarities to our public health agendas. The reason for this lies in the fact that today we face similar problems to those present in Štampar's time.

Box 1 **Andrija Štampar’s principles (13)**

1. It is more important to enlighten the people than to impose the laws; therefore, the medical profession consists of only three short laws.

2. It is most important to prepare the ground in a certain sphere and to develop the right understanding for questions of hygiene.

3. The question of public health and its improvement must not be monopolized by medical authorities, but has to be cared for by everybody, for only by joint work can the progress of health be obtained.

4. First of all, the physician must be a social worker; by individual therapy he cannot attain much, social therapy is the means of success.

5. Economically the physician must not be dependent on his patient, because it hinders him in the accomplishment of his principal tasks.

6. In matters of national health, no difference is to be made between the rich and the poor.

7. It is necessary to form a health organization, in which the physician will seek the patient, not the patient the physician; for this is the only way to gather an ever-increasing number of those whose health we have to care for.

8. The physician has to be the teacher of the people.

9. The question of national health is of a greater economic than humanitarian importance.

10. The principal fields of action of a physician are human settlements and not laboratories and consulting rooms.

In this short essay, we analyze the *Proposal for a New European Consensus on Development – Our World, our Dignity, our Future* (in further text: *Proposal*) to find out whether Štampar’s principles are still valid in the light of the SDG or if there is a need for a new approach to complement them. Although the *Proposal* encompasses a wider area than public health, it still reflects the contemporary approach to public health rooted in the SDH and SDG agenda.

The item 29 of the *Proposal*: *“The EU and its Member States will support inclusive life-long learning and equitable quality education at all levels – early childhood, primary, secondary, tertiary, technical and vocational training and adult learning, with special attention to education and training opportunities for girls and women. They will work harder to ensure everyone has the knowledge, skills, capabilities and rights they need to enjoy a life in dignity, to be fully engaged in society as responsible and productive adults, and to contribute to the social and economic well-being of their communities and to the promotion of and access to culture*.” can be linked to the first and eighth Štampar’s principle. Although today, as part of health promotion intersectorality, the role of physicians as teachers has slightly diminished, health education (or “health literacy” as we call it today) is an important part of the efforts to improve the social determinants of health (8-10).

A. Štampar’s second principle deals with social understanding and policy making when it comes to health. The same idea is present in the item 4.2. of the *Proposal,* entitled *“Fostering Stronger, More Inclusive Multi-Stakeholder Partnerships.”* For example, the item 69 reads as follows:

“*The achievement of most of the SDGs will also depend on the active involvement of local authorities. The EU and its Member States will support decentralisation reforms, when appropriate, to empower local authorities for better governance and development impact. They will support processes to help people to interact effectively with local government at all stages of policy planning and implementation*.”

The ideas stated in Štampar’s third principle agree with those in the *Proposal*. The greatest part of this document deals with issues wider than health (man-made disasters together with natural disasters, poverty, conflicts and migration), shedding light on their detrimental impact on health and society (11,12). Therefore, the approach to combating these issues should definitely be inter-sectorial and multi-sectorial.

In this context physicians should follow Štampar’s fourth principle: “*First of all the physician must be a social worker; by individual therapy he cannot attain much, social therapy is the means of success.”* and Štampar’s tenth principle: “*The principal fields of action of a physician are human settlements and not laboratories and consulting rooms.”*

Therefore, when it comes to health, the item 28 of *the Proposal* clearly states: *“Better health is the cornerstone of human dignity and global prosperity. The EU and its Member States will continue to act to strengthen health systems, prevent and combat communicable diseases, such as HIV/AIDS, tuberculosis, malaria and hepatitis, help secure affordable medicines and vaccines for all, and address global health threats, such as antimicrobial resistance. They will reduce child and maternal mortality and malnutrition, promote mental health and address the growing burden of non-communicable diseases in partner countries.”*

This approach to health can only be achieved in the light of Štampar’s seventh principle.

The authors of the *Proposal* concur with these views in the item 25: “*The SDGs highlight the areas where continued progress is required to ensure human development and dignity. The EU and its Member States will pursue an end to hunger, universal health coverage, universal access to quality education and training, adequate and sustainable social protection and decent work for all, within a healthy environment. They will support partners in fulfilling their responsibility to strengthen their national policies and governance for the sustainable provision of essential services. They will put a strong focus on the protection of the most vulnerable.”*

The issues of poverty and economic disparities chime profoundly with the ideas outlined in the *Proposal* as well as with A. Štampar's ideas. In his fifth principle he clearly stated, *“Economically the physician must not be dependent on his patient, because it hinders him in the accomplishment of his principal tasks,*” adding in the sixth principle: “*In matters of national health no difference is to be made between the rich and the poor*,” and finally concluding in the ninth principle: “*The question of national health is of a greater economic than humanitarian importance.”*

The authors of the *Proposal* are on the same page with him in the item 24: “*Eradicating poverty in all its dimensions, tackling discriminations and inequalities and leaving no one behind will remain at the heart of EU development cooperation policy, building on the major new impetus which the 2030 Agenda gives to these objectives. Progress in these areas will provide a stronger foundation for sustainable development.”* and item 30: *“Economic growth is lasting and more beneficial to the poorest, when it is inclusive. The EU and its Member States will act to reduce inequality of outcomes and opportunity.”*

Although written in different times, Štampar’s principles are based on the common values of a just society, equity, and solidarity, which are still important for public health today. These values are present in the agenda of the SDGs and *Health for All* policy by the WHO. Štampar himself encouraged those who adapted his principles to their own social context and referred to his principles as guidelines. The SDGs and WHO’s *Health for All* policy indeed used these principles as a basis, combing them with the contemporary challenges. Štampar did not support the philanthropic vision of public health still championed today by some non-governmental organizations but wanted a health system based on clear objectives, with a systematic approach to reaching these objectives. He also knew that the blue-print of his health care system organization ideas can only be successfully implemented by the unification of ideas, politics and communities, and features of countries' governance (13,14). The fact that professional public health has been formulating the same principles over and over again during the last hundred years points to the significance of the values that drive us and the difficulties we face when we try to implement these principles.
